# Shining a Light on Spectrophotometry in Bacteriology

**DOI:** 10.3390/antibiotics13121164

**Published:** 2024-12-03

**Authors:** Veronica Montesinos-Cruz, Greg A. Somerville

**Affiliations:** School of Veterinary Medicine and Biomedical Sciences, University of Nebraska-Lincoln, Lincoln, NE 68588-0905, USA; veromcpuma@gmail.com

**Keywords:** spectrophotometry, light scattering, bacterial quantitation, antibiotic resistance, persistence

## Abstract

Spectrophotometry is widely used in biological sciences. In bacteriology, spectrophotometric monitoring of cell numbers during cultivation provides a rapid assessment of growth. Unfortunately, familiarity with this technique has led scientists to become complacent in its usage. Here, we review some guiding principles of spectrophotometry and practical considerations that may influence the outcome of experiments. This perspective is intended to assist both new and seasoned scientists in presenting robust and reproducible growth data based on spectrophotometric readings.

## 1. Introduction

Bacteriologists often rely on controlled cultivation conditions to generate growth data. As growth data are often a foundational piece of information, the quality of the data are paramount for laboratory experiments, industrial fermentation processes, and many other microbiological applications. In addition, institutions such as the Clinical and Laboratory Standards Institute (CLSI; https://clsi.org/), which sets quality control standards for antimicrobial susceptibility testing, require accurate cell density determinations for minimum inhibitory concentrations (MICs) testing. There are several ways to determine the number of bacteria in a culture, such as assessing colony-forming units (cfu/mL) on solidified media or microscopically counting cell suspensions using a Petroff–Hausser counting chamber. However, the most common method involves spectrophotometry. A spectrophotometer exposes a sample to electromagnetic radiation of a defined wavelength (λ) and records the absorbance or transmittance. UV/visible spectrophotometry measures the amount of light within the UV (200–400 nm) or visible (400–700 nm) spectrum that penetrates through a sample. As incident light (input light intensity; I_o_), passes through the sample’s path length (L), some is absorbed or scattered by the sample, reducing the amount of light output (I) that is detected by a photosensor. The absorption of light by a sample depends on λ, L, and the nature of the sample (i.e., the molar concentration of the absorbing compound (c) and its chemical properties). The chemical properties of the absorbing compound are represented by a molar attenuation coefficient (ε; also known as, molar extinction coefficient) that is empirically determined for each λ (ε_(λ)_) and has units of M^−1^ cm^−1^. The relationship between absorbance (A) and concentration is defined in the Beer–Lambert Law [1,2,3] (Equation (1)), which states that:

A = c × ε_(λ)_ × L (1)

The relationship between the Beer–Lambert Law and the absorbance determined using a spectrophotometer is delineated as:

log_10_ (I_o_/I) = c × ε_(λ)_ × L (2)

For a deeper understanding of the mathematical derivations associated with these laws, theories, and concepts, the reader is directed to other excellent resources [4,5].

**Figure 1:**
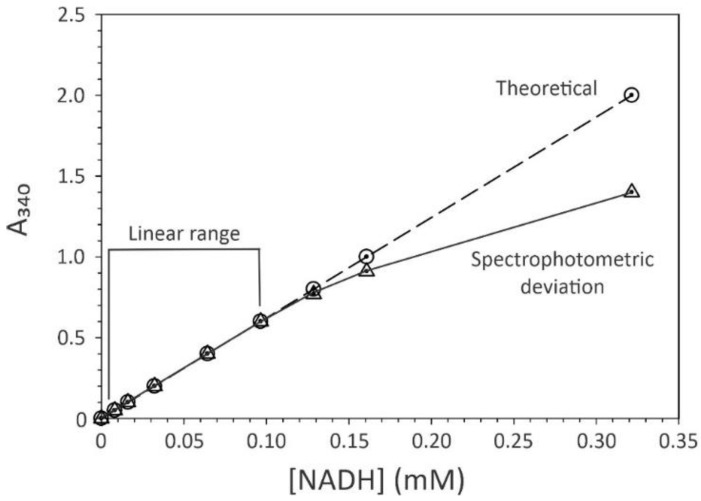
The relationship between absorbance and concentration. In this example, NADH is used as the absorbing compound at a λ = 340 nm, having a ε_(340)_ = 6220 M^−1^ cm^−1^, and an L = 1 cm. The circles with a dashed line represent the theoretical relationship between absorbance and concentration, while the triangles with a solid line demonstrate the effect of increasing concentrations on the actual absorbance. The concentration range where absorbance is proportional to the concentration of the absorbing species is defined as the linear range. All figures were created from this perspective, and not reproduced from other sources.

When determining the absorbance of pure chemical compounds, ε and L are constant; hence, the absorbance is proportional to the concentration, resulting in a linear relationship (Figure 1). In practice, this linear relationship only holds true for a narrow range of concentrations. As the concentration of an absorbing compound increases, the amount of light reaching the photosensor decreases logarithmically. For example, with nothing impeding the input light beam (i.e., I_o_ = I) a spectrophotometer’s photosensor may detect 10,000 light units. Thus, an absorbance of one, means that the light penetrating the sample (I) decreases to 1000 light units. Similarly, at two absorbance units, the light penetrating the sample decreases to 100 light units. When the absorbance is plotted as a function of concentration, this results in a saturation curve (Figure 1). In addition to scattering, stray light (i.e., when light reaches the photosensor without passing through the sample cuvette) can alter the absorbance at high concentrations or turbidities [6]. To get the actual absorbance back into the linear range, it is necessary to reduce the concentration of the absorbing compound. This relationship between concentration and absorbance also holds true when bacteria are the “absorbing” compound.

In the spectrophotometric determination of bacterial densities (also known as, turbidity, and biomass), the nature of the sample is that bacteria are particles [7]; hence, the change in “absorbance” detected by the photosensor is primarily due to the scattering of light, not the absorption of light. Light scattering is broken into two types based on the size of the scattering particles. Specifically, particles smaller than the wavelength of light being used and those close to, or larger than, the wavelength of light. For very small particles (e.g., molecules), the light wave alters the charges within a particle or molecule to the frequency of the light waves, resulting in a reradiation/scattering of light, a process called Rayleigh scattering [8]. When the size of the particle is similar to the wavelength of light and the particles are uniform spheres, the Mie theory of scattering applies [9]. The sizes of bacteria are closer to the wavelengths used in spectrophotometry and bacteria are non-uniform in size, so neither theory adequately defines bacterial light scattering. Considering these limitations, the Rayleigh–Debye–Gans theory on optically soft particles (i.e., when the refractive index of the particles, e.g., bacteria, and the surrounding medium are similar) provides a reasonable approximation of bacterial light scattering [10,11,12]. When the light scattering properties of optically soft particles (i.e., bacteria and mitochondria) were examined, the cell densities (turbidity) were found to be within a variance of 10–15% from the predicted cell densities [11,12]. From this brief summary of light scattering, it is apparent that the Beer–Lambert Law is useful for understanding the linear range of a spectrophotometer, but bacterial “absorbance” is more complicated.

**Figure 2:**
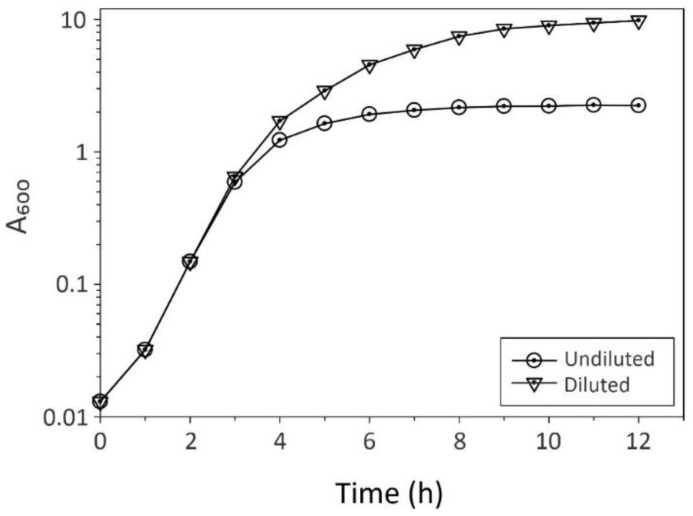
The effect of dilutions on determining the bacterial cell density over time. *Staphylococcus aureus* strain Newman was cultivated in tryptic soy broth without dextrose at 37 °C, with a flask-to-medium ratio of 10:1 and 225 rpm of aeration. Samples were harvested at the indicated times and the absorbance was assessed at a λ = 600 nm. When working with other microbial species (e.g., fungi or viruses), other wavelengths of light are more appropriate. Open circles represent culture samples whose absorbance was measured without dilution. Upside-down triangles represent samples that were assessed using dilutions (i.e., 1:5, 1:10, 1:20, or 1:50).

**Figure 3:**
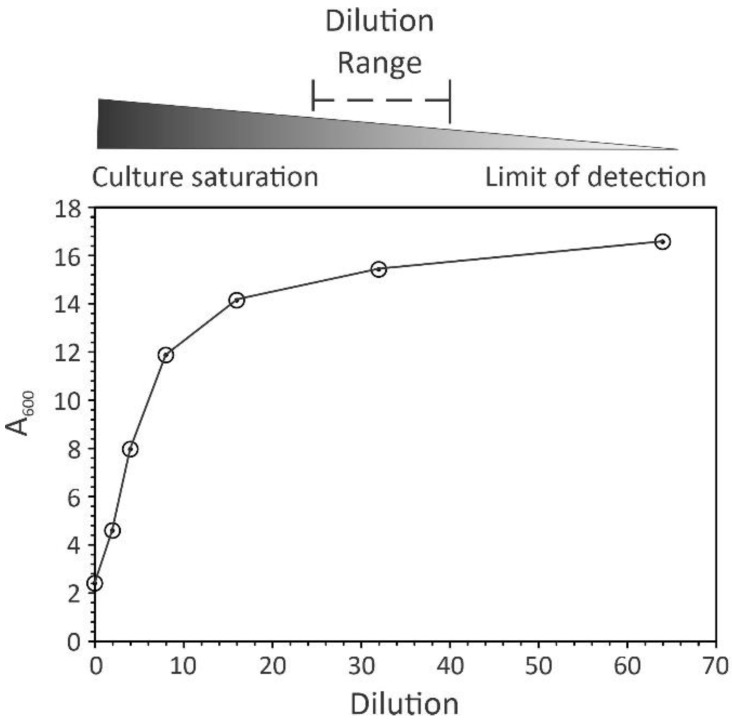
Empirical determination of appropriate bacterial culture dilutions. *Staphylococcus aureus* strain Newman was cultivated overnight in tryptic soy broth at 37 °C. Two-fold serial dilutions were prepared by diluting the overnight culture into tryptic soy broth and the absorbance was assessed at a λ = 600 nm. After measuring the absorbance of the diluted sample, the real absorbance (*Y*-axis) was determined by multiplying the diluted absorbance by the dilution factor (*X*-axis). The gradient represents an optimal dilution range between culture saturation and the photosensor’s limit of detection.

When there is a high density of bacteria in a culture, multiple scattering events occur (this is reviewed nicely in [7]), resulting in an underestimate of bacterial numbers (Figure 2) [13]. To minimize multiple scattering events, dilutions of bacterial cultures are needed to produce a concentration that will yield a preponderance of single scattering events. The cell density at which dilutions of bacterial cultures are needed is dependent on the optics and sensitivity of the spectrophotometer; hence, this must be empirically determined for each spectrophotometer (Figure 3). In addition to being dependent upon the spectrophotometer, the point at which dilutions of cultures are needed is also contingent upon the bacterial genera and species and the growth phase(s) being studied. Obviously, the shapes and sizes of different bacterial genera vary significantly, which alters their scattering properties. In addition, bacteria that form clusters tightly bound by extracellular polymers will have apparent sizes considerably larger than the individual bacteria, introducing greater variation into their light-scattering properties. Similarly, antibiotics can induce morphological changes, such as errors of cell division that create clusters or chains of bacteria. Bacterial sizes also vary as a function of the growth phase, with rapidly dividing bacteria being larger in size than quiescent bacteria. All of these factors can influence the ability to reliably determine cell numbers using a spectrophotometer and will influence the timing and magnitude of dilutions necessary for accurate growth data.

As mentioned, the bacterial growth phases can affect the accurate determination of cell numbers, but they can also affect antibiotic susceptibility. Inoculating cultures with bacteria from different growth phases alters the length of lag phases and doubling times [14]. A slower bacterial doubling time increases tolerance to antibiotics, which can be mistaken for antibiotic non-susceptibility [15]. The age of the inoculating culture can also alter the metabolic state of the bacteria, affecting antibiotic efficacy [16]. For all these reasons, a rigorous approach to monitoring bacterial growth using spectrophotometry is required in many bacteriological investigations, such as antibiotic susceptibility testing.

## 2. Practical Considerations

(1). The first factor in establishing a spectrophotometric method for following bacterial growth is to determine the wavelength of light. A wavelength of 600 nm is commonplace [17] and it minimizes the real absorbance of bacteria and culture medium, leaving light scattering as the only source of light attenuation. (2). Establish the reference standard whose absorption value will be subtracted from that of the cultivation samples. It is a general practice for microbiologists to use the cultivation medium. (3). Determine the frequency of culture sampling. This variable is dependent upon the bacterial growth rate, medium composition, and cultivation conditions [18,19]. A good rule of thumb is one sampling for every two doublings (Figure 2). For example, *Staphylococcus aureus* cultures (doubling every 24–28 min) in a nutrient-rich medium under aerobic conditions can be sampled every 60 min. Similarly, *Escherichia coli* growing in a rich medium (doubling time = 20–30 min [20]) can be sampled every 40–60 min. Slower-growing bacteria, such as *Mycobacterium tuberculosis* (doubling every 13 to 24 h [21,22]), may require sampling once every 1–2 days. When employing cultivation conditions or culture media that decrease or increase the bacterial growth rate, the sampling frequency should be changed accordingly. (4). Identify when to begin making culture dilutions to minimize multiple scattering events and maintain the absorbance in the “linear” range of the spectrophotometer (Figure 3). As mentioned, this must be determined empirically for each spectrophotometer. (5). When monitoring bacterial growth using a microplate reader, the absorbance of bacterial cultures that can achieve high cell densities will be underestimated, particularly in later growth phases [7]. For this reason, if these growth data are important for the conclusions of a manuscript, then it is appropriate to assess growth in batch cultures or fermenters where suitable dilutions can be made.

## 3. Conclusions

In the Information Age, the number of resources available to understand almost any subject is overwhelming, including the spectrophotometric determination of bacterial cell densities [13]. This abundance of knowledge can lead to information overload, which makes it more difficult to make informed decisions [23]. The goal of this perspective is to distill down a vast area of knowledge into a manageable amount of information and practical suggestions that can facilitate reliable spectrophotometric determination of bacterial cell densities. The integration of these considerations into spectrophotometry protocols facilitates a reasonable assessment of bacterial growth.

## Data Availability

No new data were created or analyzed in this study.

## Abbreviations

A = absorbance or optical density; c = molar concentration; ε = molar attenuation coefficient; ε_(λ)_ = molar attenuation coefficient at wavelength (λ); λ = wavelength; L = path length in cm.
